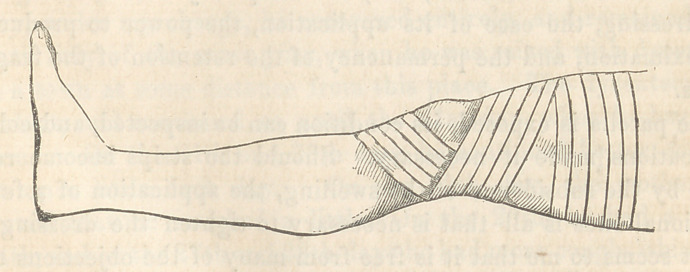# On the Use of Adhesive Plaster in the Treatment of Fracture of the Patella

**Published:** 1854-01

**Authors:** Jno. Neill

**Affiliations:** Surgeon to the Pennsylvania Hospital


### MEDICAL EXAMINER.

NEW SERIES.—NO. CIX.—JANUARY, 1854.

### ORIGINAL COMMUNICATIONS.

*On the use of Adhesive Plaster in the treatment of Fracture of the Patella.* By JNO. NEILL, M. D. Surgeon to the Pennsylvania Hospital.

From the Notes of Dr. JAMES DARRACH, House Surgeon.

The frequency of fracture of the patella and the difficulty attendant upon its treatment, are sufficient grounds for calling attention to any plan of dressing which is supposed to have some claim to utility as well as to novelty.

The necessary separation of the fragments in transverse fractures by muscular contraction, the great force necessary to produce coaptation, and the inability to maintain it without inducing great pain and sometimes excoriation, are points with which every one is familiar who has had the management of injuries of this kind.

Until a comparatively recent period it was supposed impossible to produce a bony union of the patella, and that there was something peculiar in the tissue of this bone that prevented it; therefore some of the older surgeons made little or no effort to approximate the fragments. Even at the present day, although the possibility of a perfect bony union has been demonstrated over and over again, there are some who consider the pain consequent upon the use of a retentive apparatus, and the excoriation liable to occur from tight bandaging, as too serious evils to be endured when the chances are so slight for a bony union.

It is unquestionably true, that many cases occur in which it is impossible to effect a bony union; when the periosteum is completely torn and the fragments separated for a great distance, and the soft parts contused considerably, perfect consolidation is not to be expected; at the same time it should be borne in mind, that the closer the approximation the shorter will be the ligamentous connection and consequently the more perfect will be the use of the limb subsequently, and therefore under all circumstances an effort should be made to approximate the fragments, provided it can be done in a manner which is effectual, comfortable and without risk to the patient.

A retrospect of the various means employed to carry out the well known indications in this accident will be not an inappropriate preliminary to the treatment employed in the two following cases.

Malgaigne has classified the different modes of treatment under three heads, which will be perhaps as easy a mode as any other of giving a clear and succinct account of the matter.

The *First*, includes all means which effect bony union by producing complete immobility of the limb.

Position is the only means recommended by some distinguished writers on the subject. Paul of Egina and Ambrose Paré contented themselves simply by *extending* the limb and maintaining it in that position.

Elevation was not contemplated by J. L. Petit in any other light, than that of effecting extension and favoring venous return, although he recommended the leg to be placed on a pillow. It was in 1772 that Valentine clearly announced that simple *extension* was insufficient and that *elevation* was necessary to overcome the contraction of the rectus muscle, and directed the heel to be elevated as high as possible. This he accomplished by numerous pillows placed under the thigh and leg, and three cords fastened to a slipper and reaching to the body of the patient. Richerand used merely the pillows but rejected the slipper and cords. Sabatier having observed that extreme extension produced pain in the ham, directed the knee to be slightly flexed, and having placed the leg on a pillow to the corners of which strings were attached, he suspended the limb by fastening the strings to the curtain rods. Finally, Sheldon, in 1789, insisted that the simple extended position was insufficient, because he had found by experiment that there was a difference of 2½ inches in the length of the rectus muscle in a tall person, dependent upon the horizontal or vertical position of the body, and his advice to keep the trunk in a somewhat vertical or inclined position has been followed by most English Surgeons and also by Langenbeck. Desault was the first to apply a splint to the under surface of the limb, reaching from the heel to the buttocks.

But in addition to position, many kinds of apparatus have been used to effect the more complete apposition of the fragments.

*Those acting by circular compression.*—Albucasis used an apparatus of this kind. It was a circular splint with a hole in it sufficiently large for the fragments of the patella, to which it was secured by a bandage. This was modified by Guy de Chauliac, J. De Vigo and Bassuel, and was in use as late as the latter part of the 18th century at the Hotel Dieu. Purman used a ring made of twisted iron wire and covered with leather. The *pileolus* of Meilom, a little cap stuffed with cotton, and the wooden cup of Kaldschmidt, acted on the same principle.

*Those acting by parallel compression.*—The first machine of this kind was constructed by a mechanic of Leyden, named Muschenbroek, described by Solinger, reported in France by Blein, and afterwards copied by Arnaud, who gave to it his name. It consisted of a hollow splint of iron or tin placed under the ham, and two concave plates, one of which was placed above, and the other below the patella. The edges of the splint were perforated, and corresponding holes existed in the plates, and by means of screws they were retained firmly in the proper position. This is the type of numerous machines subsequently constructed by Bucking, Evers, Böttcher, Aitken, Lampe, Morgridge, Mayor, &c., in which the shape of the splint is modified, and the material employed, wood or leather; or, the modification consists in altering the shape and material of the plates and the manner in which they are secured. Vidal uses a splint made of iron wire. The uniting bandage was first used by Hiester, and adopted by Larrey and Dupuytren, and is still seen in use; but the yielding of the bands which composed it, renders it the most unreliable of all applications.

*Those acting by concentric compression.*—The origin of this kind of apparatus is the figure of 8 bandage, applied by a roller with two heads, and described by Lavauguyon. A simple roller applied in this form with the addition of compresses above and below the fragments, has many advocates, but when used alone is liable to give by the stretching of the bandage. But the fig. of 8 employed with a straight or curved hollow splint is much more secure, and is the basis of the apparatus of Ravaton, d'Allouel, de Boyer, de Buirez, d'Assalini, &c. Boyer's apparatus, which has been much used, consists of a straight, hollow splint or long box, on the sides of which were placed nails with large heads, for the purpose of securing the padded straps which were used in place of the bandage. Recently Velpeau has returned to the fig. of 8 bandage, which he applies as a starch bandage.

*Those acting only on the upper fragment.*—This is the principle of Pott, who considered the lower fragment as essentially immovable. And all the more complicated apparatus of B. Bell, Böttcher, A. Cooper, Amesbury, &c., carry out this idea. Bell and Amesbury, nevertheless, act slightly on the lower fragment by a strap surrounding the fragment below it, but the great object of the apparatus is to bring down the upper fragment. Bell acted on the upper fragment by a long strap fastened to the shoe. Böttcher made this strap surround the foot like a stirrup. Sir A. Cooper applied above the superior fragment a leather bracelet, which could be tightened by buckles, and from one side of this bracelet there descended a long strap under the sole of the foot, which was then attached to the other side of the bracelet. He also recommended another arrangement consisting of a leather bracelet above and below the fragments, and these to be drawn together by bandages on each side.

The *Second* mode has in view the prevention of the stiffness of the knee, and contemplates a mobility of the joint long before the union is firm. This method of treatment takes its origin in England about the middle of the last century. Warner speaks of it in 1754 as being adopted by most of the London surgeons. Camper introduced it into Holland, and Flajani into Italy.

The *Third* plan of treatment may be called the mixed method. Solingen, according to the report of Camper, although he advocated the close approximation of the fragments, nevertheless recommended the flexion of the knee occasionally to prevent anchylosis. Bromfield, still more prudent, delayed the application of the apparatus until all inflammation had subsided, and commenced flexion at the end of the third week. B. Bell, on the contrary, applied the apparatus early, and made passive motion as soon as the twelfth day, and repeated it every other day. Ravaton, carefully holding the fragment in situ, made flexion at the 25th day. In addition, we may mention the apparatus of Malgaigne, which consists of two double hooks, one of which is placed in each fragment, and then they are approximated by a screw. I shall now give my own plan of treatment.

Case I. Samuel Edwards, a colored man, aged 30, was admitted into the Pennsylvania Hospital, Aug. 1st, 1853. The patella had been fractured by a severe fall from the rail of a ship, and the upper fragment was separated from the lower by a distance of an inch and a half. The contusion was so severe that great pain and swelling followed, which required leeches and cold applications, and no approximation was attempted until two weeks after the injury.

On the 17th of the month the treatment by adhesive plaster was commenced in the following manner. The plaster was cut in strips about $\frac{3}{4}$ of an inch in width. The first strip was applied by its middle immediately above the upper fragment, and the ends were firmly brought obliquely downwards and under the knee. After applying two or three more in the same way, so as fully to secure the upper fragment, then a few strips of the plaster were applied immediately below the lower fragment, as is represented in the woodcut which was sketched from the patient by Dr. Darrach.

By repeating the application of the strips alternately above and below the knee, the fragments were made to approach each other quite closely.

The muscles of the thigh were compressed by a few strips of plaster $1\frac{1}{4}$ in breadth, which were applied circularly. A short hollow splint of binder's board was then applied to the ham and retained by a roller. The limb was elevated and the trunk placed in an inclined position. This treatment was continued until October 14th, when the strips were removed. Until this time I had hoped that the union would have been bony, notwithstanding the severe nature of the injury, but was disappointed in this respect. He was discharged from the hospital, November 14th, at which time his knee-joint was perfectly moveable, and he walked without limping. The fragments were separated about one-third of an inch.

Case II.—John Morgan, aged 40, was admitted September 9th, 1853, with transverse fracture of the patella, from being violently thrust out of a house and falling on the pavement.

There was much swelling and tenderness of the knee, and the fragments were separated an inch and a half. The limb was elevated and cold lotions applied for ten days, until the inflammation had subsided. The strips were then used, but on account of the great tenderness of the parts, pressure could not be borne as well as in the preceding case.

The strips were removed on November 4th.

Nov. 18th. He was permitted to move about with the aid of a cane.

Nov. 29th. He was discharged with considerable stiffness of the knee, and fragments separated about three-fourths of an inch.

The advantages to be derived from the use of plaster in the treatment of this fracture, are, the simplicity and comfort of the dressing, the ease of its application, the power to produce approximation, and the permanency of the retention of the fragments.

The patella is exposed, its condition can be inspected, and cold applications made if necessary. Should the strips become relaxed by the subsidence of the swelling, the application of a few additional ones is all that is necessary to tighten the dressing; and it seems to me that it is free from many of the objections to which other applications are liable on the ground of complication, pain, excoriation and relaxation: and that it is particularly suitable in cases of comminuted fractures of the patella.

After these cases had been treated, and whilst the materials for this paper were being collected, I found that I had been anticipated in a great measure, if not entirely, in this mode of dressing, as will be seen by the following quotations:

"Jai vu également appliquer le huit de chiffre par M. Gama, à l'aide de moyens plus simples encore et bien antrement puissants. Au lieu de bandes ordinaires, M. Gama se sert de tres-longues bandelettes de sparadrap, qui, une fois appliquées sur les compresses graduéls, ne laissent pas cette crainte de relâchement, qui subsiste dans l'appareil dextriné, au moins jusqu' à l'entière consolidation du bandage; et de plus permettent de laisser la rotule à découvert, et de resserrer ou relâcher la pression suivant le besoin." Malgaigne, *Traité des Fractures et des Luxations,* Paris, 1847, p. 764.

"The apparatus may be very simple: the writer has generally used strips of plaster of about an inch in breadth and a foot long, crossing obliquely from the integuments immediately above the patella to the upper and back part of the leg, the patella being within the angle formed by the crossing. This, he has believed, rendered the bandage less liable to slip; but he *does not consider the plaster essential.** A moderate sized compress has been then placed immediately above the patella, the ends bending down on each side, so that the bandage has rested upon it, and has produced an equable and steady, though moderate compression, in a direction opposite to that of the extensor muscles, thereby counteracting any contraction which, under the previously detailed circumstances, they may be likely to exert. A narrow, double headed, &c., &c."—*Practical Observations on Fractures of the Patella and of the Olecranon.* By Thomas Alcock, F. R. S., p. 296.

* These italics are our own.

## Figures and Tables

**Figure f1:**